# Supplementary material for: Asynchronous magnetic resonance elastography: Shear wave speed reconstruction using noise correlation of incoherent waves
Source: Magn Reson Med. 2022 Oct 27;89(3):990–1001. doi: 10.1002/mrm.29502 (PMC9792433; doi:10.1002/mrm.29502)
Supplement: Supplementary file 1 — DATA S1 MATLAB code used to generate the numerical simulations of Figure 1 [file MRM-89-990-s002.zip › k-Wave/helpfiles/fourierShift.html]

fourierShift :: Functions (k-Wave)


# fourierShift

Resample data using a Fourier interpolant.

## Syntax

```
data = fourierShift(data, shift)
data = fourierShift(data, shift, dim)
```

## Description

`fourierShift` resamples the input `data` along the dimension `dim` using a regular grid that is offset by the non-dimensional distance `shift`. The resampling is performed using a Fourier interpolant. This function can be used to shift the acoustic particle velocity recorded by the first-order simulation functions to the regular (non-staggered) temporal grid by setting `shift` to 1/2.

```
% define input function
dt      = pi/5;
t       = 0:dt:(2*pi - dt);
y       = cos(t);

% shift by 1/2 a grid point spacing
y_shift = fourierShift(y, 1/2);

% calculate exact function for reference
y_ref   = cos(t + dt/2);

% plot comparison
plot(t, y, 'k-s', t, y_shift, 'b-s', t, y_ref, 'rx');
legend('Original Function', 'Shifted Function', 'Reference', 'Location', 'north');
```

## Inputs

|  |  |
| --- | --- |
| `data` | input data |
| `shift` | non-dimensional shift, where 0 is no shift, 1/2 is for a staggered grid, and 1 is a full grid point |

## Optional Inputs

|  |  |
| --- | --- |
| `dim` | dimension over which the signals vary in time (default = highest non-singleton dimension) |

## Outputs

|  |  |
| --- | --- |
| `shifted_data` | shifted data |

## See Also

`gradientSpect`
